# Characterization of the frameshift c.515dupC knock-in mouse model of HSPB8-associated myopathy (MFM13) and evaluation of Trehalose as autophagy-modulating therapy

**DOI:** 10.64898/2026.08.04.742148

**Published:** 2026-08-09

**Authors:** Alyaa Shmara, Lan Weiss, Anastasia Gromova, Barbara Tedesco, Pallabi Pal, Genie Kostalnick, Victoria Boock, Elizabeth Bassett, Sebastian Parera, Cheng Cheng, Lac Ta, Jonathan Lee, Arjun Panchagatti, Eshanee Mohanty, Jillian Vu, Albert R. La Spada, Angelo Poletti, Virginia Kimonis

## Abstract

Heat shock protein family B member 8 (HSPB8) is a chaperone involved in the chaperone-assisted selective autophagy (CASA) complex. HSPB8 in conjunction with cochaperone BAG3, promotes autophagy-mediated removal of misfolded proteins associated with various neurodegenerative diseases. Mutations in *HSPB8* , previously associated with Charcot Marie Tooth disease type 2L, have recently been linked to an autosomal dominant rimmed vacuolar myopathy (MFM13), and is considered a multisystem proteinopathy. Patients have distal and proximal limb girdle myopathy with muscle biopsy showing fatty replacement, endomysial fibrosis, and rimmed vacuoles leading to muscle atrophy and early demise. We have demonstrated reduced expression of HSPB8, altered autophagy and TDP-43 accumulation in patient fibroblasts. Using CRISPR technology, we generated a knock-in *Hspb* 8 mouse model of the c.515dupC hot spot frameshift variant to study disease pathology. Overexpressed murine *Hspb8* frameshift mutant (c.515dupC, fs) displays insolubility and aggregation propensity in Murine Neuroblastoma X Spinal Cord 34 (NSC-34) cells. Mutant *Hspb8* mice developed late-onset muscle weakness beginning at 15 months. Muscle biochemical analyses revealed reduced HSPB8 levels, increased TDP-43, and altered autophagy markers, partially recapitulating the human phenotype. Fiber type analysis, neuromuscular junction integrity, and motor neurons show mild myopathy without neurodegeneration. Given the lack of available treatments, we evaluated trehalose, a natural disaccharide that induces HSPB8 and enhances autophagy. Administration of 2% trehalose in drinking water improves motor performance, restores HSPB8 expression, and ameliorates autophagic and TDP-43 pathology in mutant mice. These findings support the value of our preclinical models for translational studies, and autophagy enhancement as a potential therapeutic strategy for HSPB8-related myopathy.

## Full Text Availability

The license terms selected by the author(s) for this preprint version do not permit archiving in PMC. The full text is available from the preprint server.

